# Validation of MyHPVscore: A High-Performance Human Papillomavirus Circulating Tumor DNA Laboratory-Developed Test

**DOI:** 10.1016/j.jmoldx.2026.04.003

**Published:** 2026-05-04

**Authors:** Jordan Currie, Heather Walline, Colleen G. Hochfelder, Chandan Bhambhani, Collin V. Brummel, Erin Sandford, Apurva Bhangale, Rawan Akhdar, Marisa Buchakjian, Keith A. Casper, Steven B. Chinn, David Forner, Kelly M. Malloy, Jennifer L. Shah, Andrew G. Shuman, Chaz L. Stucken, Matthew E. Spector, Francis P. Worden, Pratyusha Yalamanchi, Molly Heft Neal, Paul L. Swiecicki, Muneesh Tewari, Michelle L. Mierzwa, Mark E. Prince, J. Chad Brenner

**Affiliations:** ∗ Department of Otolaryngology–Head and Neck Surgery, Michigan Medicine, Ann Arbor, Michigan; † Cellular and Molecular Biology Program, University of Michigan Medical School, Ann Arbor, Michigan; ‡ Hematology/Oncology Division, Department of Internal Medicine, University of Michigan Cancer Center, Ann Arbor, Michigan; § Rogel Cancer Center, University of Michigan, Ann Arbor, Michigan; ¶ Department of Radiation Oncology, University of Michigan Cancer Center, Ann Arbor, Michigan

## Abstract

As rates of human papillomavirus-positive (HPV^+^) oropharynx cancer increase, there is increasing need for accurate biomarkers for diagnosis, treatment, and surveillance. The analytical performance of MyHPVscore, a droplet digital PCR laboratory-developed test, was characterized to detect circulating tumor DNA from multiple high-risk HPV (hrHPV) types (16, 18, 31, 33, 35, and 39) in plasma from patients with HPV^+^cancer. Using Clinical and Laboratory Standards Institute guidelines, MyHPVscore was developed and validated in a Clinical Laboratory Improvement Amendments–certified laboratory. Analytical controls and plasma samples from patients with HPV^+^oropharyngeal squamous cell carcinoma and noncancer controls were used to evaluate sensitivity, specificity, linearity, and analyte stability. MyHPVscore demonstrated high analytical performance for detecting multiple hrHPV types. Limits of blank were 2.7 (HPV16) and 2.6 (other hrHPV types) positive droplets/reaction; limits of detection were 15.2 and 20.8 positive droplets/reaction, respectively. The linear range was 15 to 62,500 targets/mL plasma (HPV16) and 12 to 100,000 targets/mL (other hrHPV types) with strong correlation (*R*^2^ > 0.99). Analytes were stable for 96 hours at –20°C and yielded consistent qualitative results after >400 days of storage. No cross-reactivity was observed between hrHPV types or low-risk types (HPV6 and HPV11). These results support the use of MyHPVscore as a laboratory-developed test for detecting HPV^+^oropharynx cancer. This method establishes an approach and standard controls that can benchmark other emerging circulating tumor DNA assays.

Human papillomavirus (HPV) is a common oncogenic driver of squamous cell carcinomas (SCCs) arising in the oropharynx, anus, cervix, and penis. The incidence of most HPV-positive (HPV^+^) cancers, such as HPV^+^oropharyngeal squamous cell carcinoma (OPSCC), has increased over the past two decades.^1^, ^2^, ^3^ HPV^+^cancers are expected to continue increasing in incidence for the foreseeable future despite the existence of effective multivalent vaccines.^4^ SCC of the anus, of which 90% of cases are associated with HPV, has increased in incidence by 2.7% per year from 2001 to 2015, with recent observations of particularly sharp increases in age groups >50 years old, young Black men, and patients living with HIV/AIDS.^5^ Similarly, OPSCC incidence increases 2% to 3% annually, driven primarily by an increasing proportion of HPV^+^cases,^6^ with greatest increases observed in White males.^6^^,^^7^ Although cervical cancer screening measures have been effective at reducing both the incidence and mortality of HPV^+^cervical cancer,^8^ there are no accepted means of screening for other HPV^+^cancers in the general population. Developing effective screening and cancer management tools for these diseases requires highly sensitive and specific biomarkers.

In recent years, liquid biopsy technology for monitoring HPV circulating tumor DNA (ctDNA) has shown promise as a biomarker of early recurrence (termed biochemical recurrence) in the post-treatment surveillance setting.^9^ Although several groups have advanced assays for ctDNA monitoring in the research setting,^10^ only one assay is commercially available as a laboratory-developed test for patients with HPV^+^cancer,^11^ and most studies completed to date with this assay have been performed in the setting of HPV^+^OPSCC. This assay, which detects circulating tumor tissue–modified HPV DNA, demonstrated a favorable negative predictive value when used for post-treatment surveillance.^12^^,^^13^

A single-probe droplet digital PCR (ddPCR) assay for HPV16 ctDNA was originally developed,^14^ and it was used to gauge the potential of monitoring HPV ctDNA to support the clinical management of patients with HPV^+^OPSCC. Using this initial assay, HPV ctDNA was reported to have potential for real-time monitoring of treatment efficacy and response for both primary^15^ and metastatic disease,^14^ as well as identification of biochemical recurrence during post-treatment surveillance.^16^ These results were consistent with data emerging from other groups at the time.^17^, ^18^, ^19^, ^20^ However, challenges were also noted with early single-probe assays in detection of minimum residual disease, which would have a major impact on the performance of the assays in cancer early detection and post-treatment surveillance, where high sensitivity and specificity are critical for optimal assay performance. Therefore, a high-performance HPV assay was established through development of a multiprobe ddPCR assay.^21^ This new assay demonstrated approximately ninefold increased signal when compared with the original single-probe assay. Accordingly, this new multiprobe assay was able to detect biochemical recurrence (HPV16 ctDNA) approximately 20 months earlier than the single-probe assay in index cases.^21^

Given HPV^+^cancer can result from additional high-risk types (HPV 16, 18, 31, 33, 35, and 39), there was a critical need to expand this test to include the common high-risk types beyond HPV16. After validating the improved performance of the HPV16 multiprobe assay, the assay, now called MyHPVscore, was expanded to be more comprehensive by including primers and probes targeting five additional high-risk HPV (hrHPV) types (HPV18, HPV31, HPV33, HPV35, and HPV39). On the basis of the critical need for rigorous clinical validation of HPV ctDNA through orthogonal assays in the field, this work describes the validation of the MyHPVscore assay in accordance with the Clinical Laboratory Improvement Amendments of 1988 regulations for laboratory-developed tests, such that results from the assay can be made readily available to patients and providers. The analytical results of this validation are reported here.

## Materials and Methods

### Assay Characteristics

The methods for developing the single-plex HPV16 assay were previously described.^21^ Here, the same approach was used to expand the HPV16 assay [formerly known as ctDNA HPV16 Assessment using Multiple Probes (CHAMP-16)]^21^ to generate a new assay known as the MyHPVscore assay, where an additional well is used to detect the other hrHPV types (HPV18, HPV31, HPV33, HPV35, and HPV39). Briefly, to design a multitarget assay for additional HPV strains, commonly referenced HPV variants were selected (X05015.1 for HPV18, J04353.1 for HPV31, M12732.1 for HPV33, X74477 for HPV35, and M62849.1 for HPV39), and the genomic sequences were screened computationally for candidate primers and probes. Primer/probe combinations were checked for homology to the human genome using National Center for Biotechnology Information Basic Local Alignment Search Tool (BLAST; *https://blast.ncbi.nlm.nih.gov/Blast.cgi*, last accessed July 12, 2023), as well as potential homodimerization, heterodimerization, and hairpin formation, as described previously.^21^ All sequences are listed in Supplemental Table S1.

### Validation Specimens

All patients who provided specimens provided written informed consent under studies that were approved by the University of Michigan Institutional Review Board (IRBMED number 2002-0691). Recruitment, sample collection, surveillance, and plasma collection using Cell-Free DNA BCT (Streck, La Vista, NE) or PAXgene Blood ccfDNA (PreAnalytiX, Hombrechtikon, Switzerland) tubes designed for cell-free DNA (cfDNA) preservation were previously described.^22^

### Spike-In and Engineered Controls

Positive controls and control mixes were prepared with synthetic hrHPV target sequences in HPV 16, 18, 31, 33, 35, and 39, as well as synthetic sequences specific to *RPP30* human genomic DNA and a nonhuman oligonucleotide used as a process control.^21^ The synthetic oligonucleotides were obtained from Integrated DNA Technologies (Coralville, IA) (Supplemental Table S1). Noncancer human genomic DNA and UM-SCC-104 (commercially available at Sigma, St. Louis, MO) HPV16^+^ cell line DNA controls were isolated and sheared using a Covaris (Woburn, MA) system before use in validation studies, as described previously.^21^

### Droplet Digital PCR

ddPCR was performed by preparing primer probe mixes for each assay (each primer at 16.2 μmol/L, and probes at 4.5 μmol/L), and subsequent reaction mixes with 2× ddPCR Supermix (no dUTP), sample or control template, and the primer probe mix (each primer is 438.8 nmol/L, and probes are 122.9 nmol/L in the final reaction mix). The Bio-Rad (Hercules, CA) QX200 system was used for droplet generation (Automated Droplet Generator), thermal cycling, reading the droplets, and data analysis.

### Limit of Blank, Limit of Detection, and Limit of Quantitation

Limit of blank (LOB), limit of detection (LOD), and limit of quantitation (LOQ) were defined on the basis of Clinical and Laboratory Standards Institute guidelines. Blank wells [Qiagen (Hilden, Germany) Buffer EB] were monitored over a period of 2 years and assayed for HPV16 and other hrHPV types. The limit of the blank was defined as follows:

(1) $$\text{LOB}={\text{mean}}_{\text{blank}}+1.645\left({\text{SD}}_{\text{blank}}\right)$$

For the HPV16 assay, serially diluted samples of sheared DNA extracted from a cultured HPV16^+^ cell line (UM-SCC-104) were measured in triplicate from 7 to 1000 HPV16 targets per assay well (0.4 to 55 human haploid genomic equivalents). The LOD was calculated using the LOB, as described above, and the SD of the low concentration sample with the following equation, at which 95% of values will exceed the defined LOB^11^^,^^23^, ^24^, ^25^, ^26^:

(2) $$\text{LOD}=\text{LOB}+1.645\left({\text{SD}}_{\text{low}\;\text{concentration}\;\text{sample}}\right)$$

Dilution series of pooled HPV18, HPV31, HPV33, HPV35, and HPV39 templates were tested from 10 to 100,000,000 targets/well of each high-risk type. The LOD was calculated using Equation 2 with the low concentration sample from this experiment.

Verification of the LOD was performed for both HPV assay wells. As the provisional LOD is expected to generate a result greater than the LOB 95% of the time, acceptance criteria for this experiment were detection (signal greater than the assay LOB) of HPV in at least 19 of 20 replicates. A total of 20 replicates of sheared cell line DNA (UM-SCC-104 and UM-SCC-105 for HPV16 and other hrHPV, respectively) corresponding to the calculated LOD values were tested.

For the HPV16 assay, a greater range of concentrations (30 to 125,000 targets/well) was assessed to saturate the signal and determine the upper limit of quantitation (ULOQ).

The lower LOQ and ULOQ were defined as the lowest and highest concentrations, respectively, tested that remained within the linear range of the assays. Multiple replicates at the LOQ concentration were assayed to confirm repeated measurement results in a CV of ≤20%.

Serial dilutions of cfDNA eluates extracted from HPV^+^clinical plasma samples were also measured to ensure assay linearity performs as expected with the clinically relevant sample matrix. Three samples each for HPV16 and other hrHPV types were tested in duplicate at eight dilutions.

### MyHPVscore

A final score is calculated for each assay well (HPV16 and other hrHPV types) as the sum of positive partitions in both channels minus the per-assay limit of detection.

(3) $${\text{MyHPVscore}=N}_{\text{pos}\_1}+N_{\text{pos}\_2}-LOD$$

Negative scores are reported as zero. Scores greater than zero indicate a sample is positive for the given assay.

### Specificity and Cross-Reactivity with Common Low-Risk HPVs

Assays were further tested against plasma samples prepared with synthetic low-risk HPV6 and HPV11 template oligonucleotides. Five oligonucleotides each for both HPV6 and HPV11 were designed for the same viral genomic regions assayed for high-risk HPV (*E6*, *E1*, *L2*, *L1*, and *URR*) (Supplemental Table S1). The oligonucleotides were pooled into type-specific mixes and tested at four concentrations on the MyHPVscore test to confirm that the low-risk types were not detected with the high-risk or control assays. The low-risk oligonucleotide pools were also spiked into known hrHPV^+^clinical plasma samples, which were then tested with and without the low-risk spike to confirm that the presence of other viral types would not compromise the performance of the test.

### Carryover

To verify that positive samples do not contaminate subsequent runs, a plate of water blanks was tested following evaluation of a plate of all positive samples. The predefined acceptance criteria for this test were that all water controls must be found to be below the LOD for this assay.

### Accuracy and Precision

Genomic DNA extracted from 21 known HPV^+^tumor samples, cfDNA from matched patient plasma samples, genomic DNA from 20 known HPV-negative (HPV^–^) tumor samples, and 20 cfDNA samples from plasma of patients with no history of cancer were analyzed, to assess qualitative performance. Considering that the HPV16 multiprobe assay targets nine different genomic loci and was designed for fragmented plasma cfDNA, results from formalin-fixed, paraffin-embedded (FFPE) tumor samples were not used for quantitative assessment. HPV status was confirmed by targeted capture-based next-generation sequencing (NGS) analysis using the MiOTOseq panel described in NGS Reference Assay: MiOTOseq. Total allowable inaccuracy was defined *a priori* at 12%, as recommended by guidance from the Association of Public Health Laboratories.^27^

To assess quantitative performance, plasma samples from patients with HPV^+^ (*n* = 25) and HPV^–^ (*n* = 20) tumors previously confirmed by targeted-capture sequencing were divided into two aliquots and tested in parallel with the MyHPVscore assay and a reference HPV ctDNA NGS assay described in NGS Reference Assay: Plasma. MyHPVscore testing was performed by H.W., who was blinded to HPV status. Quantitative results from both assays were compared.

The assay's analytical precision was considered by testing three plasma samples pooled from multiple HPV^+^patients a total of five times, across multiple instruments and multiple days. Total allowable imprecision was defined as <20% CV.

### Stability of DNA Analytes from Plasma

Specimen stability was tested to ensure that analyte integrity is not compromised in primary or processed samples because of storage. Human plasma was supplemented with synthetic HPV templates for both HPV assay wells. These were analyzed (including cfDNA extraction and ddPCR) at time point zero and after storage at –80°C at 24-hour intervals for 96 hours. This process was repeated for extracted cfDNA stored at –20°C for the same time durations. Three clinical plasma samples positive for HPV16 were tested in duplicate immediately after cfDNA extraction and again after 7 days of cfDNA storage at –20°C. Long-term stability was assessed using a larger cohort of both HPV16-positive and HPV16-negative clinical plasma samples subjected to cfDNA extraction and MyHPVscore testing immediately after plasma separation and again after 200 to 400 days of plasma storage at –20°C.

### NGS Reference Assay: Plasma

cfDNA samples isolated from 2 mL plasma were prepped as NGS libraries using the xGen cfDNA & FFPE DNA Library Prep v2 MC Kit with high-density capture probes covering the entire genome of all HPV types tested by MyHPVscore (Integrated DNA Technologies) used for custom capture. Sequencing was performed on an Illumina (San Diego, CA) NovaSeqX.

### NGS Reference Assay: MiOTOseq

Tumor genomic DNA was isolated, as previously described,^28^ and sequenced as with the plasma reference assay, using the capture panel described. HPV typing of NGS libraries was performed using HPViewer (*https://github.com/yuhanH/HPViewer*; commit c62f29e), following quality control and adapter trimming. FASTQ files with adequate depth were processed using default HPViewer parameters to detect and genotype HPV.^29^

Statistical analyses were conducted in R version 4.4.1 (*https://www.r-project.org*).

### Data Availability

The data generated in this study are not publicly available because of patient privacy requirements but are available on reasonable request from the corresponding author.

## Results

### Development of the High-Performance MyHPVscore ddPCR Assay

**Figure 1:**
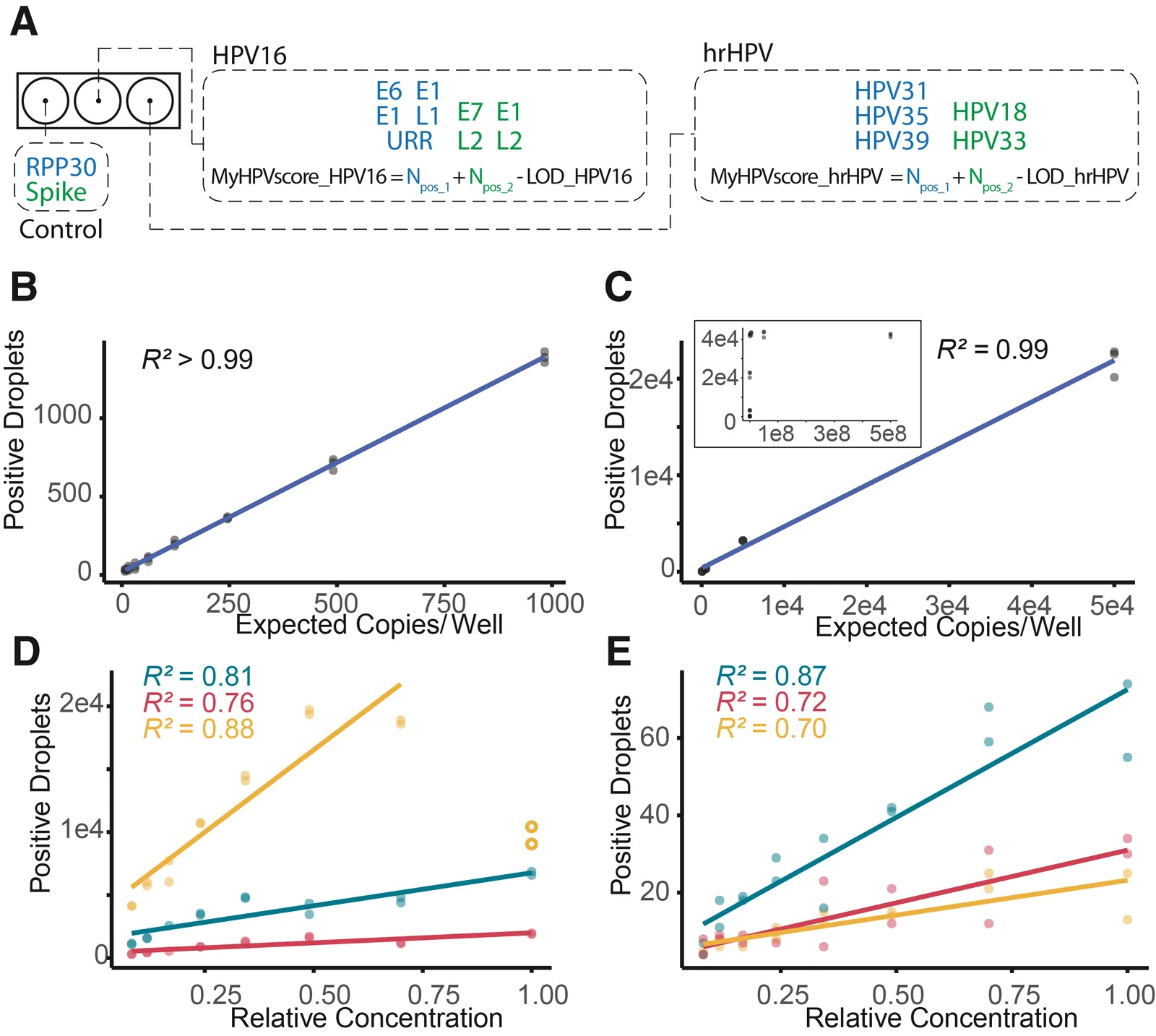
**A:** Schematic of the MyHPVscore three-well assay configuration. Well 1 tests for human gene *RPP30* and a spiked-in process control sequence. Well 2 detects nine regions of the HPV16 genome within two channels. Well 3 detects five additional high-risk HPV (hrHPV) types within two channels. A score is generated for wells 2 and 3 by summing positive partitions in each channel within a given well. **B:** Serial dilution of extracted genomic DNA from HPV16-positive cell line UM-SCC-104. Eight concentrations in a twofold dilution series were tested in triplicate, and positive droplet counts in both measured channels (from fluorescent labels FAM and VIC) were summed. All dilutions were within the linear range of the HPV16 assay. **C:** Serial dilution of synthetic hrHPV mixture. **Main panel:** Linear range. **Inset:** Entire range tested. Eight concentrations in a 10-fold dilution series were tested in triplicate. *R*^2^ value calculated only from concentrations within the selected linear range (triplicate measurements of the lowest three concentrations). **D:** The 1.43-fold serial dilution of cell-free DNA (cfDNA) extracted from three known HPV16-positive clinical plasma samples denoted by different colors. Each dilution was tested in duplicate. Open yellow circles were excluded from the regression because of saturated signal. **E:** The 1.43-fold serial dilution of cfDNA extracted from three known hrHPV-positive clinical plasma samples denoted by different colors. Each dilution was tested in duplicate. **Solid lines** represent simple linear regression, with associated *R*^2^ values included for each fitted model. LOD, limit of detection.

**Supplemental Figure S1:**
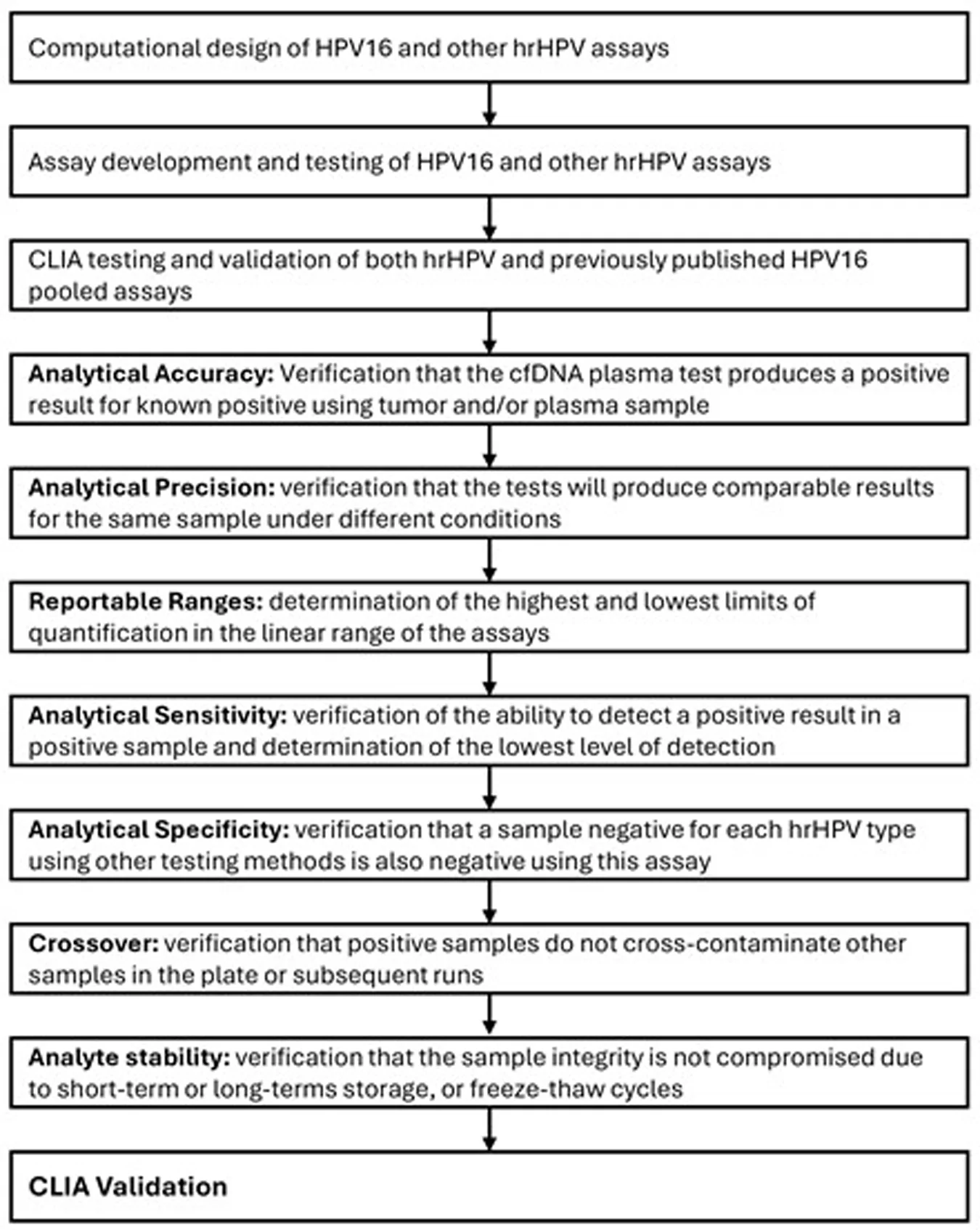
Clinical Laboratory Improvement Amendments (CLIA) validation process. cfDNA, cell-free DNA; hrHPV, high-risk HPV.

Given the clinical need for a high-performing test targeting the most common high-risk types of HPV, the existing HPV16 assay was expanded to include an additional well that detects additional hrHPV types (HPV18, HPV31, HPV33, HPV35, and HPV39) (Figure 1A). Candidate primer/probe combinations for each HPV type with optimal characteristics for a multiprobe assay were selected for further experimental screening and development following a procedure previously detailed.^21^ A final two-well HPV assay using a total of 28 primers and 14 probes was selected for Clinical Laboratory Improvement Amendments validation testing (Supplemental Figure S1). This included a pool of 18 primers and 9 probes for multiple regions of the HPV16 genome tested in one well, and a pool of single primer pairs and probes each for HPV18, HPV31, HPV33, HPV35, and HPV39 tested in another well. An additional well measured the human *RPP30* gene and a nonhuman sequence spiked into samples as an internal process control.

### Molecular Pathology Approach to Analytical Validation

LOB, LOD, and LOQ were defined on the basis of Clinical and Laboratory Standards Institute guidelines and are summarized in Table 1.

**Table 1:** LOB, LOD, LLOQ, and ULOQ for Each Assay Well

Assay well	LOB	LOD	LLOQ	ULOQ
HPV16	2.7	15.2	21.9	21,500
Other hrHPV	2.6	20.8	29.5	21,800

Values are reported as total positive droplets.

hrHPV, high-risk HPV; LLOQ, lower limit of quantitation; LOB, limit of blank; LOD, limit of detection; ULOQ, upper limit of quantitation.

#### Limit of the Blank

The LOB is defined as the highest number of positive droplets that might be found in repeated measures of a blank and was calculated with Equation 1. Blank wells (Qiagen Buffer EB) measured for HPV16 and other hrHPV types over a 2-year period were retrospectively reviewed, for a total of 203 wells per assay, giving LOB values of 2.7 and 2.6 positive droplets for HPV16 and other hrHPV types, respectively.

#### LOD and Linear Range

For the HPV16 assay, serially diluted samples of sheared DNA extracted from a cultured HPV16^+^ cell line (UM-SCC-104) were measured in triplicate from 0.43 to 55 haploid genomic equivalents per well. In our hands, this cell line has been measured to have two copies of HPV16 per human genomic equivalent (data not shown) and would be expected to contain 18 targets per human genomic equivalent with the nine-probe assay (Figure 1B). The entire dilution series was within the linear range of the assay.

The LOD for HPV16 was calculated with Equation 2 using the LOB and the low concentration sample from Figure 1B, resulting in an LOD of 15.2 positive droplets per reaction.

When defined as in Equation 2, the calculated LOD was expected to exceed the defined LOB 95% of the time. For verification of this value, also known as LOD_95, 20 replicates of sheared UM-SCC-104 (HPV16^+^) DNA corresponding to the calculated LOD_95 were tested. All 20 wells were positive for HPV16 (mean = 10.95 positive droplets; SD = 2.35; 21.5% CV).

The HPV16 LOQ was verified by taking repeated measurements (*n* = 20) of a sample at the lowest point in the linear range of the assay (0.043 human genomic equivalents, and 0.76 HPV16 targets/μL), with an average result of 21.9 (SD = 2.13; 9.7% CV) positive droplets, equivalent to 15.2 targets/mL of plasma and a MyHPVscore of 6.7, calculated using Equation 3.

**Supplemental Figure S2:**
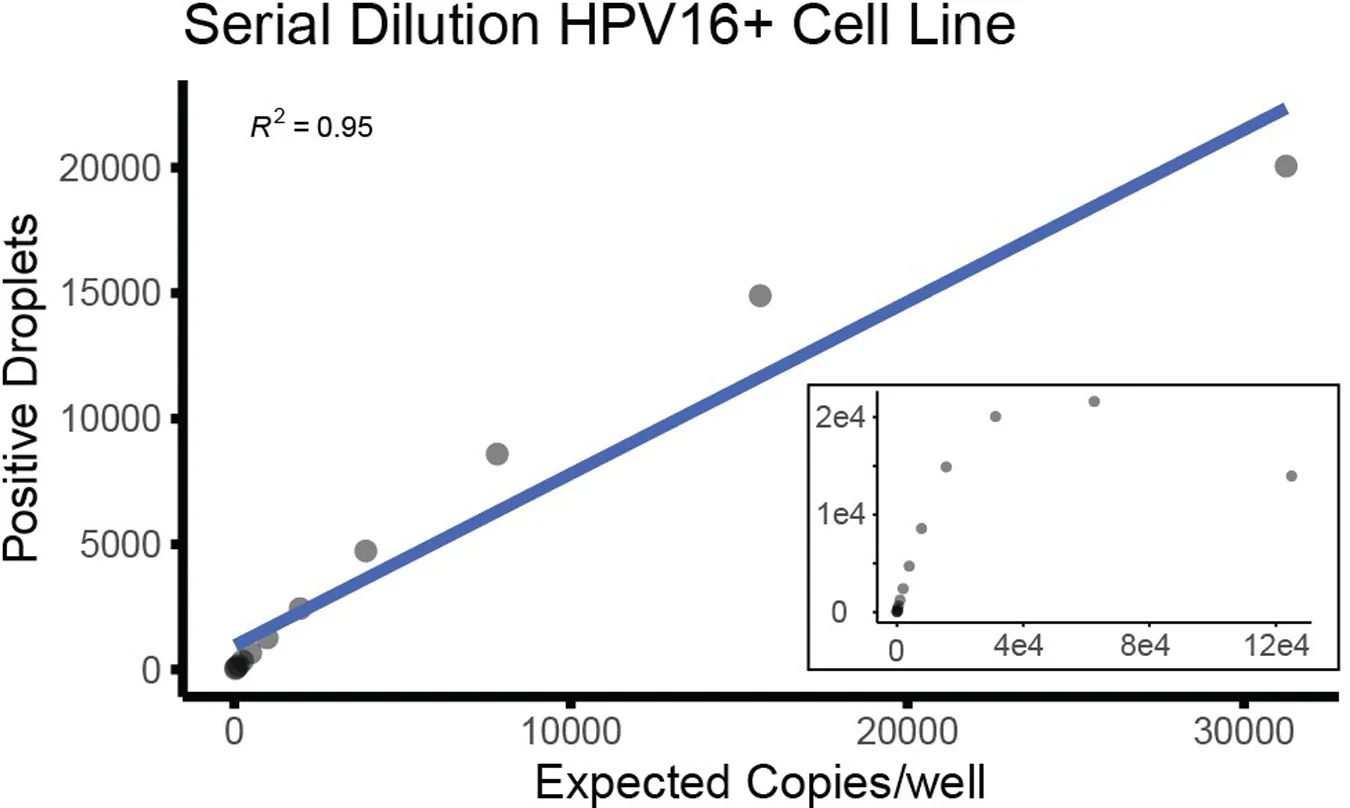
Twofold serial dilution of UMSCC104 at 13 concentrations spanning 30 to 125,000 targets per well to establish the linear range of the assay. Each point tested in singleton. **Main panel:** Linear range. **Inset:** Entire range tested. Linear model fit only to data shown in the main panel (30 to 30,000 targets/well)—points outside the linear range were excluded from the regression analysis.

A greater range of concentrations was assessed to saturate the signal and determine the ULOQ (Supplemental Figure S2). This was determined to be 21,500 positive droplets summed across both channels from 3125 HPV16 targets/μL (equivalent to 62,500 targets/mL plasma), above which HPV16 was still identified and resulted in a positive MyHPVscore but no longer scaled linearly with increasing targets.

The hrHPV assay was assessed by running a range experiment using synthetic HPV DNA template oligonucleotides (1 to 10,000,000 targets/μL each type) to define the linear range of the assay (Figure 1C). The LOD for hrHPV was assessed using the LOB and the low concentration sample from Figure 1C, resulting in an LOD_95 of 20.8 positive droplets per reaction. The ULOQ was defined as the top of the linear range, or 21,800 positive droplets summed across both channels from 1000 targets/μL of each type, equal to 50,000 targets/well. Above the linear range, hrHPV was detected resulting in a positive test, with recovery of <90% (Figure 1C). The hrHPV LOQ was determined by taking repeated measurements (*n* = 30) of a sample at the lowest point in the linear range of the assay (3 targets total per μL, averaging 0.6 targets per type/μL), with an average result of 29.5 (SD = 4.3; 15% CV) positive droplets, equivalent to a MyHPVscore of 8.7, calculated using Equation 3.

To verify the LOD_95 for hrHPV, 20 replicates of sheared UM-SCC-105 (HPV18^+^) DNA corresponding to the calculated LOD_95 were tested. All 20 wells were positive for hrHPV (mean = 17.95 positive droplets; SD = 2.76; 15.4% CV).

Assay linearity was further assessed using serial dilutions of cfDNA eluates extracted from HPV^+^clinical plasma samples (Figure 1, D and E). Undiluted cfDNA from one HPV16^+^ sample fell outside the linear range of the assay and was excluded from the regression analysis. Serial dilutions of cfDNA from clinical samples showed strong linearity (*R*^2^ values per sample range from 0.70 to 0.88), suggesting that for positive MyHPVscore values above the ULOQ, it may be possible for samples to be diluted and reanalyzed within the linear range when quantitative results are needed. This linearity was present even despite differing slopes across clinical samples, which can be caused by a combination of differing numbers of HPV genome copies per patient, HPV genomic alterations in target regions, and, in the case of non-HPV16 hrHPV, differing HPV types.

#### Specificity

**Figure 2:**
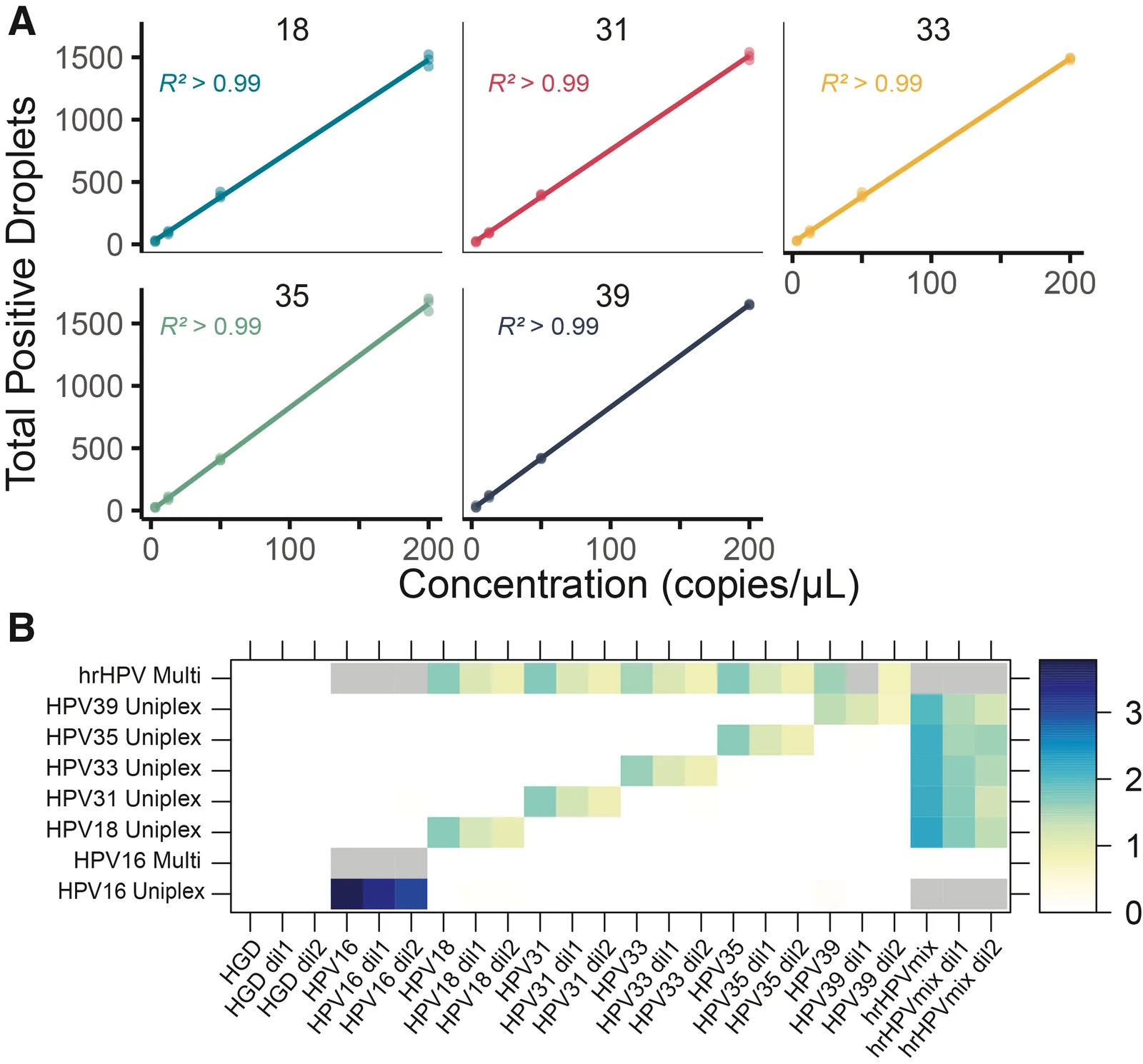
**A:** Fourfold dilution (dil) series of synthetic templates specific to each individual high-risk HPV (hrHPV) type were tested at four concentrations in triplicate with the multiprobe hrHPV assay. **Solid lines** represent simple linear regression, with associated *R*^2^ values included for each fitted model. **B:** Specificity across assays. Threefold dilution series of synthetic templates were measured with individual primer probe mixes (*y* axis: uniplex) or the final multiprobe assays (*y* axis: multi) at three concentrations each. Each assay and dilution combination presented were tested in singleton. The HPV16 and other hrHPV types assays showed no cross-reactivity among their target synthetic oligonucleotides, and neither assay amplified sheared human genomic DNA (HGD). *y* Axis: uniprobe and multiprobe assays. *x* Axis: sample. Gray cells = no data. The hrHPV multi-assay with sample HPV39 dilution 1 failed because of no accepted droplets (well failure). The remaining gray cells were not tested in this experiment, as these combinations of assays and targets were evaluated with a greater number of concentrations and replicates presented elsewhere in this article. Heat map scale represents log10-transformed positive droplet counts. White = 0.

**Supplemental Figure S3:**
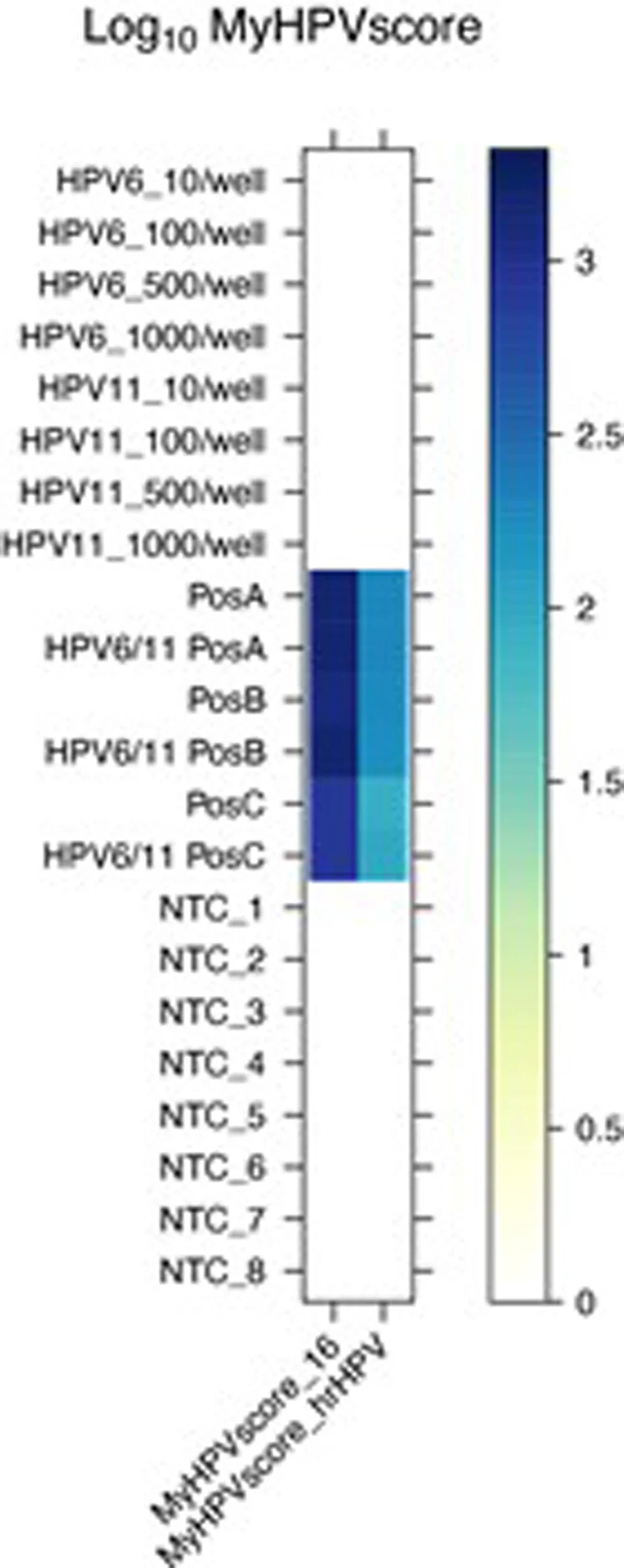
Specificity. Heat map scale represents log10-transformed MyHPVscore. HPV6 and HPV11: Four-point dilution series of synthetic templates selected to match the genomic regions tested by the two assays (*E1*, *E6*, *L1*, *L2*, and *URR*). Numbers/well represent the number of synthetic oligonucleotides included per well. PosA, PosB, and PosC: pooled positive clinical samples, pooled to be positive for both HPV16 and other high-risk HPV (hrHPV) types. HPV 6/11 PosA, HPV 6/11 PosB, HPV 6/11 PosC: same pools spiked with HPV6 and HPV11 synthetic templates. The HPV16 and other hrHPV types assays show no cross-reactivity with synthetic oligonucleotides specific to low-risk types HPV6 and HPV11. When these low-risk type oligonucleotides are mixed with the targets of the assays, amplification is not impeded. NTC, no template control.

Synthetic templates specific to each individual hrHPV type were assessed with the hrHPV assay and showed linearity within the tested range (*R*^2^ > 0.99 for all high-risk types) (Figure 2A). When tested with individual primer/probe sets or when using the final multiplexed assays, the HPV16 and other hrHPV type assays showed no cross-reactivity among their target synthetic oligonucleotides, and neither assay amplified sheared human genomic DNA (Figure 2B). Oligonucleotides for HPV6 and HPV11 were designed for the viral genomic regions assayed for high-risk HPV (*E6*, *E1*, *L2*, *L1*, and *URR*). The oligonucleotides were pooled into type-specific mixes and tested at four concentrations on the MyHPVscore test, confirming that these low-risk types were not detected with the high-risk or control assays. The low-risk oligonucleotide pools were also spiked into known hrHPV^+^clinical plasma samples, which were then tested with and without the low-risk spike-in, confirming that the presence of other viral types does not compromise the performance of the test (Supplemental Figure S3).

#### Carryover

Carryover was tested by analyzing a plate of water blanks following known positive samples. The only detected positive droplets were in the HPV16 wells (13 of 30 wells) and were below the assay LOD (range, 0 to 4 droplets; mean, 0.68 droplets; SD, 0.98 droplets). The hrHPV and control wells had 0 detected positive droplets. A positive HPV control run on the same plate detected an expected amount of positive HPV droplets and *RPP30* droplets (2688 and 48 droplets, respectively).

#### Accuracy

To demonstrate the accuracy of the qualitative assay results in clinical samples, genomic DNA extracted from HPV^+^ (*n* = 21) and HPV^–^ (*n* = 20) tumors, confirmed by targeted-capture sequencing, was tested with the MyHPVscore assay along with cfDNA extracted from patient-matched plasma samples. All samples from patients with HPV^+^tumors were positive for HPV16, and all samples from patients with HPV^–^ tumors were negative for HPV (Supplemental Table S2 and Supplemental Table S3). Beyond the qualitative results, MyHPVscore results from plasma samples did not correlate linearly with MyHPVscore from patient-matched extracted tumor DNA (*R*^2^ = 0.02) and were only modestly correlated with HPV16 read counts from targeted capture sequencing of the same tumors (*R*^2^ = 0.22). This was expected as ctDNA shedding rates are highly variable and influenced by several factors, including tumor volume, treatment status, and primary versus nodal disease. Previous work suggested that pretreatment ctDNA abundance was more closely correlated with nodal tumor volume but not primary tumor volume. Furthermore, during chemoradiation treatment, week 2 ctDNA values were significantly negatively correlated with primary tumor volume.^15^ Additionally, genomic DNA extracted from tumors was expected to generate overlapping PCR artifacts with the HPV16 mutiprobe assay because of the longer fragment size of tumor-derived DNA, which was not anticipated in plasma cfDNA because of the short nature of circulating DNA fragments. Given this technical concern and the complex relationship between tumor characteristics and ctDNA abundance, it was not surprising that there was a lack of correlation of quantitative scores between tumor and plasma, but strong correlation on qualitative calls (HPV typing).

**Figure 3:**
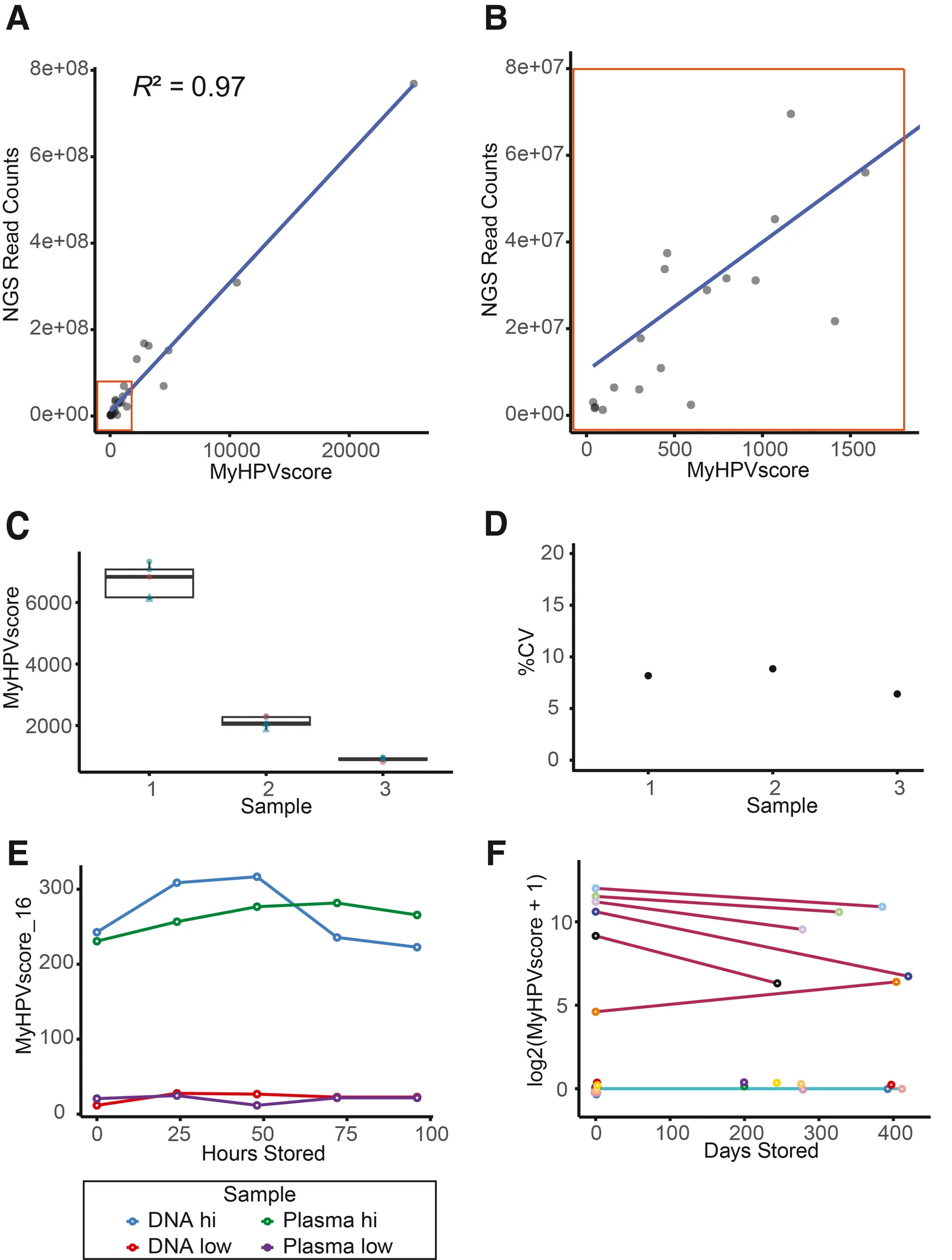
Accuracy, precision, and stability. **A:** Concordance of results of cell-free DNA (cfDNA) extracted from plasma and split between MyHPVscore and next-generation sequencing (NGS). *R*^2^ value is from linear regression on all positive samples. **B:** Same as **A**, zoomed in to the orange box. **C:** cfDNA from plasma samples positive for HPV16 was pooled and tested repeatedly over multiple plates, multiple days, and across multiple instruments. **D:** %CV from **C**. **E:** Repeated measurements of samples stored over 96 hours. Primary samples (plasma) spiked with high and low concentrations of synthetic oligonucleotides of HPV sequences were stored at –20°C and subjected to cfDNA extraction and MyHPVscore testing at each time point (plasma high and plasma low), and cfDNA extracts of the same samples (DNA high and DNA low) were stored and tested at each time point. MyHPVscore_HPV16 is shown. **F:** Long-term clinical plasma stability. cfDNA was extracted fresh from plasma at each time point. Maroon = positive results at both time points. Turquoise = negative results at both time points. Points of negative MyHPVscore samples are plotted with jitter for visibility—all have a MyHPVscore of 0.

For further validation and quantitative comparison, plasma samples from patients with HPV^+^ (*n* = 25) and HPV^–^ (*n* = 20) tumors were divided into two aliquots and tested in parallel with the MyHPVscore assay and a reference HPV ctDNA NGS deep sequencing assay. The MyHPVscore plasma assay demonstrated 100% concordance with the reference HPV ctDNA NGS deep sequencing assay across the 45 samples, with perfect sensitivity and specificity. Critically, the quantitative results were highly correlated (*R*^2^ = 0.966), supporting strong linearity and robust assay performance across clinically relevant ctDNA levels (Figure 3, A and B).

#### Precision

Analytical precision was then assessed by pooling cfDNA extracted from HPV16^+^ clinical samples and testing multiple aliquots per pool a total of five times, across multiple instruments and days. All positive samples tested positive on all five repeats, and results were highly concordant across runs, with <10% CV within all sample pools (Figure 3, C and D).

#### Stability and Recovery

**Supplemental Figure S4:**
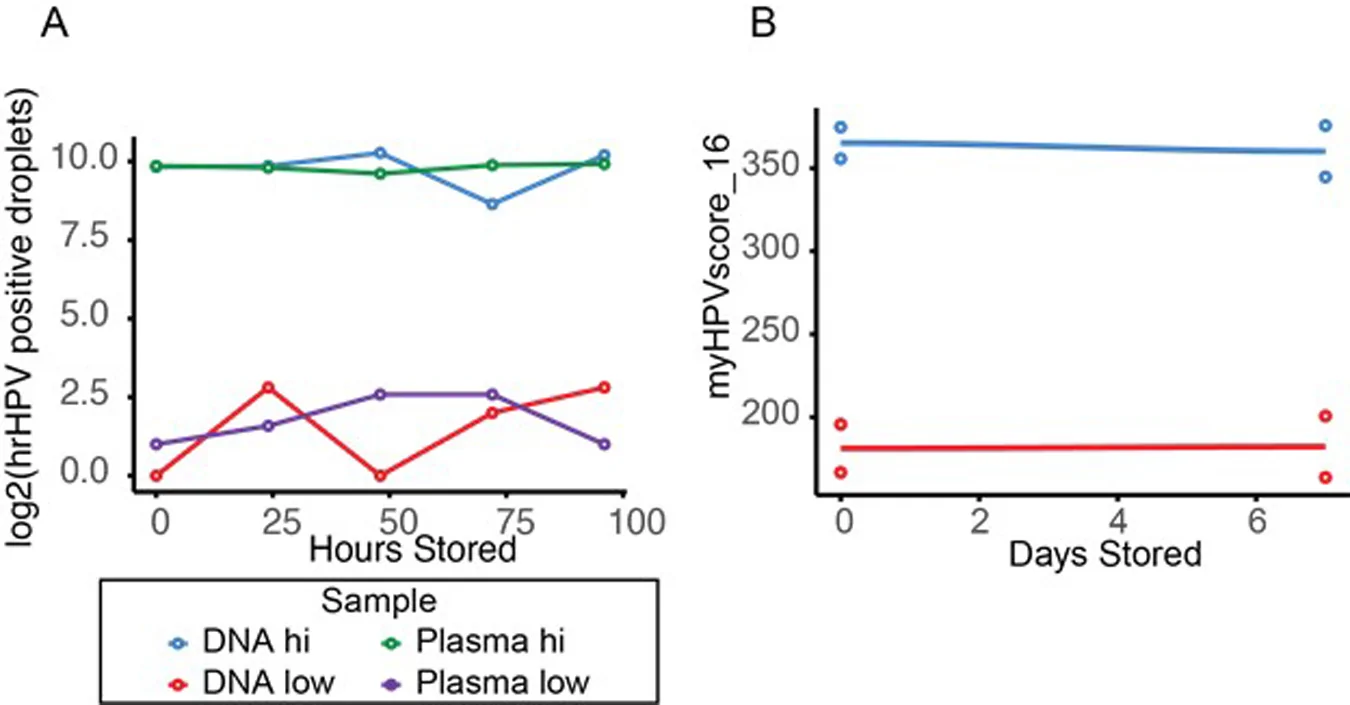
Additional stability information. **A:** Repeated measurements using the MyHPVscore other high-risk HPV (hrHPV) type assay on samples stored over 96 hours. Primary samples (plasma) spiked with high and low concentrations of synthetic oligonucleotides of HPV sequences were stored at –20°C and subjected to cell-free DNA (cfDNA) extraction and MyHPVscore testing at each time point (plasma high and plasma lo), and cfDNA extracts of the same samples (DNA high and DNA low) were stored and tested at each time point. MyHPVscore_hrHPV is shown. **B:** Processed (cfDNA) clinical samples known to be positive for HPV16 were tested in duplicate immediately after cfDNA extraction and after storage at –20°C for 7 days.

Stability of samples was assessed with repeated measurements of samples at low and high concentrations of synthetic spike-in oligonucleotides for both primary (plasma) and processed (extracted DNA) samples after being frozen at –80°C (plasma) or –20°C (extracted cfDNA) for 24, 48, 72, and 96 hours: HPV16 (Figure 3E) and other hrHPV types (Supplemental Figure S4A). Both primary and processed samples were stable through the time course tested (Figure 3E). Processed (cfDNA) clinical samples known to be positive for HPV16 were tested in duplicate immediately after cfDNA extraction and after storage at –20°C for 7 days and showed concordant results (Supplemental Figure S4B).

Finally, a large cohort of clinical samples were retested after plasma storage ranging from 200 to 420 days. Despite a reduction in score of most positive samples, suggesting some sample degradation during storage, all positive samples remained positive across the entire time range tested, and all negative samples remained negative (Figure 3F). Although this was considerably longer than the storage time for clinical specimens and clinical reporting, banked samples were of interest for longitudinal research studies and future benchmarking studies. In these cases, quantitative results may underestimate the original amount of ctDNA present in the sample, but here had reliable qualitative results across a wide range of ctDNA levels.

### Implementation of MyHPVscore in a Clinical Setting

**Figure 4:**
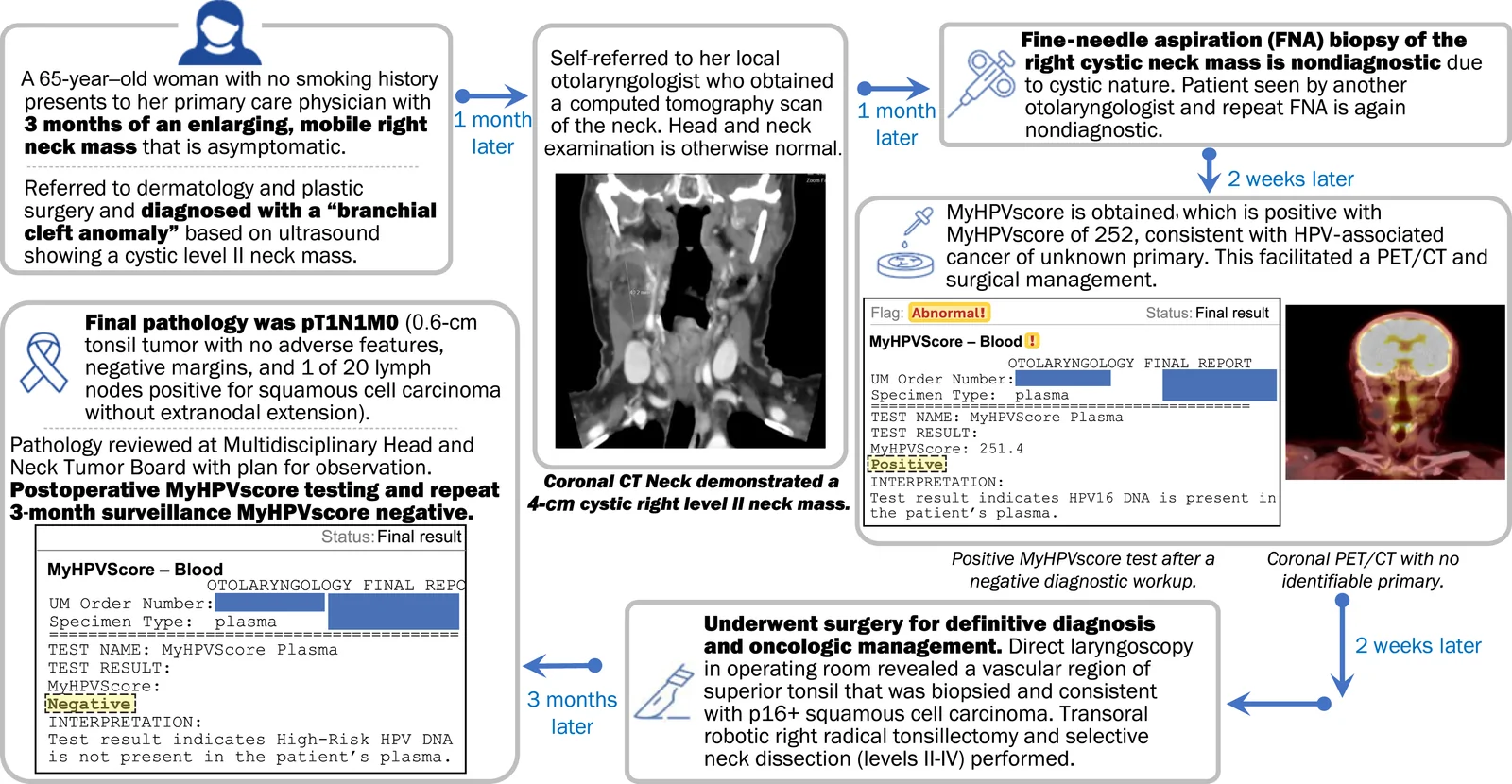
Utilization of MyHPVscore in oncologic diagnostic workup: case example of MyHPVscore informed diagnostic workup of a persistent neck mass. CT, computed tomography; FNA, fine-needle aspiration; PET, positron emission tomography.

Here, a recent case example using MyHPVscore in the diagnostic workup of the adult persistent neck mass and subsequent oncologic surveillance is presented. A 65-year-old woman with no smoking history presented with 3 months of an asymptomatic, enlarging, mobile right neck mass. Fine-needle aspiration was nondiagnostic because of the cystic nature of the neck mass, which is a common feature of HPV-related OPSCC nodal disease. Because of suspicion for malignancy, MyHPVscore was obtained, which was positive. This facilitated subsequent positron emission tomography–computed tomography (PET/CT) imaging, surgical workup to identify the primary site of disease, and definitive oncologic management, as described in Figure 4.

## Discussion

Following the initial publications by Chera et al^20^ and Haring et al,^14^ which suggested the clinical potential of HPV ctDNA, the HPV ctDNA field has made a major impact on the clinical management of patients with HPV^+^cancers. This success can largely be credited to the early deployment of HPV ctDNA testing by the Naveris team via the NavDx test.^11^ Now, the field is experiencing rapid expansion, with a variety of assays soon to become commercially available to providers, including assays built on the ddPCR platform (such as MyHPVscore and NavDx) as well as a variety of NGS-based assays.

Given the growing complexity of the HPV ctDNA testing landscape, it is critical that the field standardizes the molecular pathology approach to validating assays and establishes a set of commercially available reference controls. This work provides a stepwise workflow for analytical lockdown of HPV ctDNA assay performance, and identifies universal controls, such as the UM-SCC-104 and UM-SCC-105 cell lines, which may serve as universal reference points for assay benchmarking. Importantly, these controls were chosen in addition to synthetic DNA as they are naturally HPV integrated cell lines with comprehensive whole-genome sequencing data publicly available. This information will be critical to support future benchmarking and proficiency testing studies, where issues like HPV copy number and sequence features (eg, single-nucleotide polymorphisms) may have an impact on the interpretation of results. Furthermore, because clinical samples positive for HPV types other than HPV16 are more challenging to obtain (approximately 10% to 12% of HPV^+^cases harbor non-HPV16 hrHPV types),^30^ reference controls with alternative hrHPV types, like UM-SCC-105 (HPV18^+^), will be invaluable for assay validation protocols.

Using the evaluation workflow described, the analytical performance of the MyHPVscore ctDNA assay was comprehensively characterized. The MyHPVscore assay had comparable analytical performance to the commercially available NavDx test.^11^ Notably, the NavDx assay detects HPV16, HPV18, HPV31, HPV33, and HPV35, whereas the MyHPVscore assay is designed to detect these types with the addition of HPV39. Gunning et al^11^ reported that the characteristics of the NavDx assay were LOBs of 0 to 0.32 copies/μL, LODs of 0 to 1.10 copies/μL, and LOQs of <1.20 to 4.11 copies/μL. Direct analytical comparison between the two assays is challenging because of the differing units (copies/μL in NavDx versus positive partitions/well in MyHPVscore) and different source materials used to define the analytical performance of the assays, demonstrating the clear value of standardized controls to benchmark future assays.

As noted, the HPV ctDNA testing landscape will also soon include NGS-based assays. A recent head-to-head comparison between ddPCR strategies and HPV-DeepSeek reported significant increases in sensitivity and specificity using the NGS approach.^31^ The authors note the importance of extremely high sensitivity in a screening setting because of the low level of ctDNA at preclinical stages of disease. Their comparator ddPCR assay used one probe per HPV type, whereas the MyHPVscore assay uses nine probes targeting nine distinct regions of the HPV16 genome, and a separate pooled well of one probe each for the remaining high-risk types tested. This distinction is noteworthy, because a ninefold increase in analytical sensitivity was demonstrated with the current multiprobe assay compared with previous single-probe assays,^21^ suggesting there may be a narrower gap in clinical sensitivity between multiplex ddPCR and NGS strategies than in past comparisons. Accordingly, this study showed 100% concordance between HPV ctDNA NGS deep sequencing and MyHPVscore for baseline detection of HPV ctDNA. Further studies are necessary to clarify if potential gains in analytic sensitivity with NGS-based assays result in improved clinical sensitivity sufficient to justify increased costs, or whether NGS assays will have higher utility as a reflex test for patients following a positive ddPCR result.

As interest in HPV ctDNA biomarker use grows, there is increasing demand for clinical utility studies with HPV ctDNA assays.^32^^,^^33^ A vignette of the potential clinical utility of MyHPVscore is shown in Figure 4. Following successful analytical validation, the clinical application of this assay has been preliminarily characterized in two recently completed clinical trials. The first characterized performance during longitudinal surveillance was in A Multi-Center Phase II Trial of Individualized Adaptive De-escalated Radiotherapy Using Pre and Mid-Treatment FDG-PET/CT for HPV-related Oropharynx Cancer (NCT03416153). In this cohort of 68 evaluable patients, MyHPVscore demonstrated a strong negative predictive value, with >95% detection rate for clinical recurrence with biochemical lead time of 28 to 161 days over the first clinical detection of recurrence.^34^ The point-in-time negative predictive value >99% was reported in the trial. In the second trial, MyHPVscore absolute scores and kinetics were assessed in a clinical trial investigating the addition of nivolumab to chemoradiotherapy in high-risk OPSCC as a potential biomarker for patient outcomes,^35^ and importantly only weakly correlated with outcome features, demonstrating the need for additional data to understand how to implement ctDNA testing for real-time therapy adjustments. Advancing assays such as MyHPVscore will facilitate broader clinical access to ctDNA as a biomarker across institutions and patient populations. This increased access not only supports early detection of recurrence but also may support additional trials investigating its clinical utility as a dynamic and predictive biomarker.

The field of HPV-driven cancer is rapidly transitioning, with multiple cooperative groups considering the recommendation of HPV ctDNA testing as a supplement to post-treatment surveillance. Nonetheless, robust clinical utility data validating its use for this purpose are currently incomplete.^33^

## Conclusion

Overall, this article outlines a molecular pathology approach to standardizing the analytical characterization of emerging HPV ctDNA assays. In this work, a multiplex ddPCR assay covering high-risk HPV types responsible for >99% of HPV^+^cancers (HPV 16, 18, 31, 33, 35, and 39) was developed and analytically characterized. The MyHPVscore assay demonstrates excellent analytical performance, and early clinical trials have demonstrated strong clinical performance consistent with other published ctDNA assays.^34^^,^^35^ Although these HPV ctDNA laboratory-developed tests are already being used to complement patient care, robust clinical utility trials that compare multiple assays are still needed.^33^ The addition of the MyHPVscore assay to the clinical landscape for HPV^+^cancers brings the field one step closer to leveraging liquid biopsy technology to improve oncologic outcomes.

## Disclosure Statement

The droplet digital PCR assay presented in this study is now commercially available based on a license agreement with Bio-Rad. C.B., C.V.B., M.T., and P.L.S. may benefit financially. All other authors declare no competing interests.
