# Supplementary material for: Validation of MyHPVscore: A High-Performance Human Papillomavirus Circulating Tumor DNA Laboratory-Developed Test
Source: J Mol Diagn. 2026 May 4;28(7):553–64. doi: 10.1016/j.jmoldx.2026.04.003 (PMC13494182; doi:10.1016/j.jmoldx.2026.04.003)
Supplement: Supplementary file 1 [file mmc1.docx]

**Supplemental Table S1.** Sequences for all template oligos, primers, and probes utilized in the validation study.

| **Name** | **Sequence** | **Type** |
| --- | --- | --- |
| HPV6E6 | 5’-AATGCGGTTGTATATAAACCAGCCCTAAAATTTAGCAAACGAGGCATTATGGAAAGTGCAAATGCCTCCACGTCTGCAACGACCATAGACCAGTTGTGCAAGACGTTTAATCTATCTATGCATACGTTGCAAATTAATTGTGTGTTTTGTCACT-3’ | Template oligo |
| HPV6E1 | 5’-AATGCTCCACCTCTGCTAGTAACGTCCAACATAGATATTACTAAAGAAGATAAATATAAGTATTTACATACTAGAGTAACAACATTTACATTTCCAAATCCATTCCCTTTTGACATCACT-3’ | Template oligo |
| HPV6L2 | 5’-AATGCACATAATTCGTTTGCACAGACCTGCCATTGCGTCCCGACGTGGCCTTGTGCGGTACAGTCGCATTGGACAACGGGGGTCTATGCACACTCGCAGCGGAAAGCACATAGGGGTCACT-3’ | Template oligo |
| HPV6L1 | 5’-AATGCGGGGACAGCCATTAGGTGTGGGTGTAAGTGGACATCCTTTCCTAAATAAATATGATGATGTTGAAAATTCAGGGAGTGGTGGTAACCCTGGACAGGATAACAGGGTTAATGTAGGTATGGATTTCACT-3’ | Template oligo |
| HPV6URR | 5’-AATGCTGCAACCGTTTTCGGTTGCCCTTAGCATACACTTTCCACCAATTTGTTACAACGTGTTTCCTCTTAATCCTATATATTTTGTGCCAGGTACACATTGCCCTGCCAAGTTGCTTGCCAAGTGCTCACT-3’ | Template oligo |
| HPV11E6 | 5’-AATGCGGTTATATATAAACCAGCCCAAAAAATTAGCAGACGAGGCATTATGGAAAGTAAAGATGCCTCCACGTCTGCAACATCTATAGACCAGTTGTGCAAGACGTTTAATCTTTCTTTGCACACTCTGCAAATTCAGTGCGTGTTTTGTCACT-3’ | Template oligo |
| HPV11E1 | 5’-AATGCTCCACCGCTACTGGTTACATCAAATATAGACATTAGCAAAGAGGAGAAATACAAATATTTACATAGTAGAGTTACCACATTTACATTTCCAAATCCATTCCCCTTTGACATCACT-3’ | Template oligo |
| HPV11L2 | 5’-AATGCATATTATTAGACTACATAGACCAGCTATAACGTCCAGACGGGGTCTTGTGCGTTTTAGTCGCATTGGGCAACGGGGGTCCATGTACACACGCAGTGGACAACATATAGGTGTCACT-3’ | Template oligo |
| HPV11L1 | 5’-AATGCGGGGTCAACCTTTAGGCGTTGGTGTTAGTGGGCATCCATTGCTAAACAAATATGATGATGTAGAAAATAGTGGTGGGTATGGTGGTAATCCTGGTCAGGATAATAGGGTTAATGTAGGTATGGATTTCACT-3’ | Template oligo |
| HPV11URR | 5’-AATGCTGCAACCGTTTTCGGTTGCCCTTACATACACTTACCTCAAATTTGTTATAACGTGTTTTGTACTAATCCCATATGTTGTGTGCCAAGGTACATATTGCCCTGCCAAGTATCTTGCCAACAACTCACT-3’ | Template oligo |
| Nonhuman spike in control | 5’-TGTTTTCTTTAGTACTCAGAAAGGTTGTGTAGGATTGTTGAGAGTGTTGGAGAAGGAGTGACTGATCTTCAACCAGGAGATCATGTTTTACCCATCTTCACCGGAGAGTGTGGGGATTGTCCTCACTGTCACTCCGAGGAATCCAACATG -3’ | Template oligo |
| HPV18 | 5’-AATGCTGTGAGAAACACACCACAATACTATGGCGCGCTTTGAGGATCCAACACGGCGACCCTACAAGCTACCTGATCTGTGCACGGAACTGAACACTTCACTGCAAGACATATCACT-3’ | Template oligo |
| HPV31 | 5’-AATGCGAGGCAACTGACCTCCACTGTTATGAGCAATTACCCGACAGCTCAGATGAGGAGGATGTCATAGACAGTCCAGCTGGACAAGCAGAACCGGACACATCCAATTACAATATCGTTACTCACT-3’ | Template oligo |
| HPV33 | 5’-AATGCGATTTCATAATATTTCGGGTCGTTGGGCAGGGCGCTGTGCGGCGTGTTGGAGGTCCCGACGTAGAGAAACTGCACTGTGACGTGTAAAAACGCCATGAGAGGACACAAGCCAACGTTAAAGGTCACT-3’ | Template oligo |
| HPV35 | AGGTCGGTGTATGTCCTGTTGGAAACCAACACGTAGAGAAACCGAGGTGTAATCA-3’ | Template oligo |
| HPV39 | 5’-AATGCGAACCACAGCGTCACACAATACAGTGTTCGTGTTGTAAGTGTAACAACACACTGCAGCTGGTAGTAGAAGCCTCACGGGATACTCTGCGACAACTACAGCAGCTGTTTATGGACTCACTAGGATTTGTGTGTCCGTGGTGTGTCACT-3’ | Template oligo |
| HPV16_Seq_5 | 5’-GGAACAACATTAGAACAGCAATACAACAAACCGTTGTGTGATTTGTTAATTAGGTGTATTAACTGTCAAAAGCCACTGTGTCCTGAAGAA-3’ | Template oligo |
| HPV16_Seq_6 | 5’-GAGAGGGTGTTAGTGAAAGACACACTATATGCCAAACACCACTTACAAATATTTTAAATGTACTAAAAACTAGTAATGCAAAGGCAGCAATGTTAGC-3’ | Template oligo |
| HPV16_Seq_7 | 5’-GTAATAAATCAACGTGTTGCGATTGGTGTATTGCTGCATTTGGACTTACACCCAGTATAGCTGAC-3’ | Template oligo |
| HPV16_Seq_16 | 5’-GGCATTAGTGGCCATCCTTTATTAAATAAATTGGATGACACAGAAAATGCTAGTGCTTATGCAGCAAATGCAGGTGTGGATAATAG-3’ | Template oligo |
| HPV16_Seq_19 | 5’-AGTTCTATGTCAGCAACTATGGTTTAAACTTGTACGTTTCCTGCTTGCCATGCGTGCCAAATCCCTGTTTTCCTGACCTGCACTG-3’ | Template oligo |
| HPV16_Seq_2 | 5’-CTAACATTAATGCTGGTACAGATTCTAGGTGGCCTTATTTACATAATAGATTGGTGGTGTTTACATTTC-3’ | Template oligo |
| HPV16_Seq_4 | 5’-AGAACACGTAGAGAAACCCAGCTGTAATCATGCATGGAGATACACCTACATTGCATGAATATATGTTAGATTTGCAACCAGAGACAACTGATCTC-3’ | Template oligo |
| HPV16_Seq_13 | 5’-CTACACATAATAATCCCACTTTCACTGACCCATCTGTATTGCAGCCTCCAACACCTGCAGAAACTGGAGGGCATTT-3’ | Template oligo |
| HPV16_Seq_15 | CCAGCATTAACCTCTAGGCGTACTGGCATTAGGTACAGTAGAATTGGTAATAAACAAACACTACGTACTCG-3’ | Template oligo |
| HPV16_5_seq_F | 5’-GGAACAACATTAGAACAGCAA-3’ | Primer |
| HPV16_5_seq_R | 5’-TTCTTCAGGACACAGTGG-3’ | Primer |
| HPV16_6_seq_F | 5’-GAGAGGGTGTTAGTGAAAGA-3’ | Primer |
| HPV16_6_seq_R | 5’-GCTAACATTGCTGCCTTTGC-3’ | Primer |
| HPV16_7_seq_F | 5’-GTAATAAATCAACGTGTTGCG-3’ | Primer |
| HPV16_7_seq_R | 5’-GTCAGCTATACTGGGTGTAA-3’ | Primer |
| HPV16_16_seq_F | 5’-GGCATTAGTGGCCATCCTT-3’ | Primer |
| HPV16_16_seq_R | 5’-CTATTATCCACACCTGCATTTGC-3’ | Primer |
| HPV16_19_seq__F | 5’-AGTTCTATGTCAGCAACTATGG-3’ | Primer |
| HPV16_19_seq_R | 5’-CAGTGCAGGTCAGGAAA-3’ | Primer |
| HPV16_2_seq_F | 5’-CTAACATTAATGCTGGTACAGA-3’ | Primer |
| HPV16_2_seq_R | 5’-GAAATGTAAACACCACCAATC-3’ | Primer |
| HPV16_4_seq_F | 5’-AGAACACGTAGAGAAACCC-3’ | Primer |
| HPV16_4_seq_R | 5’-GAGATCAGTTGTCTCTGGTT-3’ | Primer |
| HPV16_13_seq_F | 5’-CTACACATAATAATCCCACTTTCAC-3’ | Primer |
| HPV16_13_seq_R | 5’-AAATGCCCTCCAGTTTCT-3’ | Primer |
| HPV16_15_seq_F | 5’-CCAGCATTAACCTCTAGGC-3’ | Primer |
| HPV16_15_seq_R | 5’-CGAGTACGTAGTGTTTGTTTATTAC-3’ | Primer |
| PV16_5_seq_FAM_F | 5’-CAACAAACCGTTGTGTGATTTG-3’ | Probe |
| HPV16_6_seq_FAM_F | 5’-CACTATATGCCAAACACCACTTAC-3’ | Probe |
| HPV16_7_seq_FAM_F | 5’-TGGTGTATTGCTGCATTTGG-3’ | Probe |
| HPV16_16_seq_FAM_F | 5’-AAATGCTAGTGCTTATGCAGCAA-3’ | Probe |
| HPV16_19_seq_FAM_F | 5’-CTTGTACGTTTCCTGCTTGC-3’ | Probe |
| HPV16_2_seq_VIC_F | 5’-TCTAGGTGGCCTTATTTACA-3’ | Probe |
| HPV16_4_seq_VIC_R | 5’-TCATGCAATGTAGGTGTATCTCC-3’ | Probe |
| HPV16_13_seq_VIC_F | 5’-CATCTGTATTGCAGCCTCCA-3’ | Probe |
| HPV16_15_seq_VIC_R | 5’-ATTCTACTGTACCTAATGCCAGTAC-3’ | Probe |
| RPP30_F | 5’-AGATTTGGACCTGCGAGC-3’ | Primer |
| RPP30_R | 5’-GGAGCGGCTGTCTCCACAAG-3’ | Primer |
| Spike_F | 5’-GATCTTCAACCAGGAGATCA-3’ | Primer |
| Spike_R | 5’-AGTGACAGTGAGGACAATCC-3’ | Primer |
| Spike_VIC | 5’-ACCCATCTTCACCGGA-3’ | Probe |
| RPP30_FAM | 5’-CCGCGCAGAGCCTTC-3’ | Probe |
| HPV31_4_F | 5’-GACCTCCACTGTTATGAGCAA-3’ | Primer |
| HPV31_5_R | 5’-CTATGACATCCTCCTCATCTGA-3’ | Primer |
| HPV35_1_F | 5’-AGGTCGGTGTATGTCCTGTTG-3’ | Primer |
| HPV35_2_R | 5’-TGATTACACCTCGGTTTCTC-3’ | Primer |
| HPV39_7_F | 5’-CGGGATACTCTGCGACAACT-3’ | Primer |
| HPV39_8_R | 5’-CACACCACGGACACACAAAT-3’ | Primer |
| HPV18_4_F | 5’-ACCACAATACTATGGCGCGC-3’ | Primer |
| HPV18_5_R | 5’-GGTAGCTTGTAGGGTCGCCG-3’ | Primer |
| HPV33_4_F | 5’-AAACTGCACTGTGACGTGTA-3’ | Primer |
| HPV33_5_R | 5’-CTTTAACGTTGGCTTGTGTC-3’ | Primer |
| HPV18_CLIA_6PrF | 5’-TGAGGATCCAACACGG-3’ | Probe |
| HPV31_CLIA_6PrF | 5’-TACCCGACAGCTCAG -3’ | Probe |
| HPV33_CLIA_6PrF | 5’-AAACGCCATGAGAGGAC-3’ | Probe |
| HPV35_CLIA_3PrR | 5’-ACGTGTTGGTTTCCAAC-3’ | Probe |
| HPV39_CLIA_9PrF | 5’-CAGCAGCTGTTTATGGACTCA-3’ | Probe |
