# Supplementary material for: Validation of MyHPVscore: A High-Performance Human Papillomavirus Circulating Tumor DNA Laboratory-Developed Test
Source: J Mol Diagn. 2026 May 4;28(7):553–64. doi: 10.1016/j.jmoldx.2026.04.003 (PMC13494182; doi:10.1016/j.jmoldx.2026.04.003)
Supplement: Supplementary file 2 [file mmc2.docx]

**Supplemental Table S2.** Result concordance between different sample types and testing modalities in HPV16-positive clinical samples.

| **ID** | **MyHPVscore - Plasma** | **MyHPVscore - Tumor** | **HPV16 Sequencing Reads - Tumor** |
| --- | --- | --- | --- |
| 5037 | 22067.7 | 23687.7 | 2298751 |
| 4854 | 10424.7 | 26332.7 | 8415182 |
| 4502 | 10286.7 | 16864.7 | 21092544 |
| 4997 | 3283.7 | 245.7 | 2097262 |
| 4862 | 3062.7 | 550.7 | 380661 |
| 5003 | 2486.7 | 35122.7 | 3674111 |
| 5002 | 1135.7 | 29303.7 | 379022 |
| 4934 | 960.7 | 7980.7 | 79886 |
| 5011 | 933.7 | 25534.7 | 2925506 |
| 4875 | 879.7 | 2155.7 | 1275129 |
| 4980 | 357.7 | 32310.7 | 531831 |
| 4736 | 333.7 | 1551.7 | 1632178 |
| 4896 | 270.7 | 37294.7 | 495950 |
| 5016 | 244.7 | 9389.7 | 1059296 |
| 4737 | 210.7 | 9080.7 | 350866 |
| 4082 | 148.7 | 31453.7 | 429561 |
| 5000 | 127.7 | 28139.7 | 278659 |
| 4703 | 90.7 | 13373.7 | 24107 |
| 4945 | 85.7 | 9894.7 | 10460 |
| 4687 | 62.7 | 15318.7 | 3803641 |
| 5015 | 41.7 | 974.7 | 324917 |
